# Unlocking the Potential of the Human Microbiome for Identifying Disease Diagnostic Biomarkers

**DOI:** 10.3390/diagnostics12071742

**Published:** 2022-07-19

**Authors:** Rima Hajjo, Dima A. Sabbah, Abdel Qader Al Bawab

**Affiliations:** 1 Department of Pharmacy, Faculty of Pharmacy, Al-Zaytoonah University of Jordan, P.O. Box 130, Amman 11733, Jordan; dima.sabbah@zuj.edu.jo (D.A.S.); abdelqader.albawab@zuj.edu.jo (A.Q.A.B.); 2 Laboratory for Molecular Modeling, Division of Chemical Biology and Medicinal Chemistry, Eshelman School of Pharmacy, The University of North Carlina at Chapel Hill, Chapel Hill, NC 27599, USA; 3 National Center for Epidemics and Communicable Disease Control, Amman 11118, Jordan

**Keywords:** biomarkers, diagnostic biomarkers, metagenomics, microbial metabolites, microbiome, microbiome biomarkers, microbiota biomarkers

## Abstract

The human microbiome encodes more than three million genes, outnumbering human genes by more than 100 times, while microbial cells in the human microbiota outnumber human cells by 10 times. Thus, the human microbiota and related microbiome constitute a vast source for identifying disease biomarkers and therapeutic drug targets. Herein, we review the evidence backing the exploitation of the human microbiome for identifying diagnostic biomarkers for human disease. We describe the importance of the human microbiome in health and disease and detail the use of the human microbiome and microbiota metabolites as potential diagnostic biomarkers for multiple diseases, including cancer, as well as inflammatory, neurological, and metabolic diseases. Thus, the human microbiota has enormous potential to pave the road for a new era in biomarker research for diagnostic and therapeutic purposes. The scientific community needs to collaborate to overcome current challenges in microbiome research concerning the lack of standardization of research methods and the lack of understanding of causal relationships between microbiota and human disease.

## 1. Introduction

The human microbiota comprises 10–100 trillion symbiotic microbial cells constituting over 10,000 microbial species residing in the human body and outnumbering human cells by 10 times [1]. It consists primarily of bacteria, in addition to viruses, fungi, protozoa, and helminths residing in and on human body organs, such as the skin, mammary glands, mucosa, gastrointestinal (GI), respiratory, and urogenital tracts [2,3,4]. The largest percentage of the human microbiota (95%) resides in the GI tract, and every human being has a unique microbiota composition which could potentially serve as a unique fingerprint. The human microbiome consists of the genes of prokaryotic and eukaryotic cells, and it is often viewed as our “other genome”, which consists of more than three million genes, in comparison with our 23,000 human genes. Hence, the human microbiome has gained increased interest recently with regard to identifying novel drug targets and biomarkers for human disease.

Microbiota affect human health and disease by modulating important metabolic and immunomodulatory processes [3,5]. The interactions between the human body and microbiota form a complex, distinct, and harmonized bionetwork that defines the relationship between the host and its microbiota as commensal, symbiotic, or pathogenic. The human microbiota is continually developing and changing throughout life by responding to host factors such as age, genes, hormonal changes, nutrition, predisposing disease, lifestyle, and many environmental factors [6,7,8,9]. Harmonized microbiota contribute substantially to healthy livelihood [7], while a disruption in microbiota hemostasis (dysbiosis) might contribute to life-threatening diseases [10]. The significant contribution of the human microbiome in health and disease has been recently described in the biomedical literature [11,12,13,14,15,16,17,18,19,20,21,22] delineating gastrointestinal [10,23,24,25,26,27,28,29,30,31,32,33,34,35,36,37], urinary tract [4,38], and skin [3] microbiota. Evidence from the biomedical literature indicates that alterations in host immunity might be closely related to the compositional and functional changes of gut flora [24,39]. 

Thus, the human microbiota can potentially lead to the discovery of effective disease diagnostic biomarkers. According to the National Institute of Health (NIH), a biomarker is “a characteristic that is objectively measured and evaluated as an indicator of normal biologic processes, pathogenic processes, or pharmacologic responses to a therapeutic intervention” [40]. A diagnostic biomarker is simply a biomarker that “detects or confirms the presence of a disease or condition of interest, or identifies an individual with a subtype of the disease” [41]. The most frequently used biomarkers are derived from either biological materials or imaging data. More recently, machine learning (ML) and artificial intelligence (AI) have enabled the identification of highly predictive, disease-specific biomarkers [42]. 

In fact, microflora disturbances have been linked to many human diseases, including GI tract diseases [10,43], cardiovascular disease [13,44,45], allergies [39,46], inflammation [44,45,47], neuro-disease, stubborn bacterial infections [48,49,50,51], and cancer [37,52,53,54,55,56,57,58,59,60,61,62,63,64,65,66,67,68]. Aberrations in the human microbiome are linked to several cancers, including breast, colorectal, gastric, pancreatic, and hepatic cancers [69,70]. Additionally, cancer could be provoked by viruses, fungi, helminths, and bacteria [69,70]. Microbiota might also contribute to cancer development by disrupting the balance between the growth and death of host cells after altering the immune system and affecting metabolism [58,71,72]. Furthermore, Microbiota affects cancer prognosis by several mechanisms, including genotoxicity, inflammation, and metabolism [73]. 

**Figure 1:**
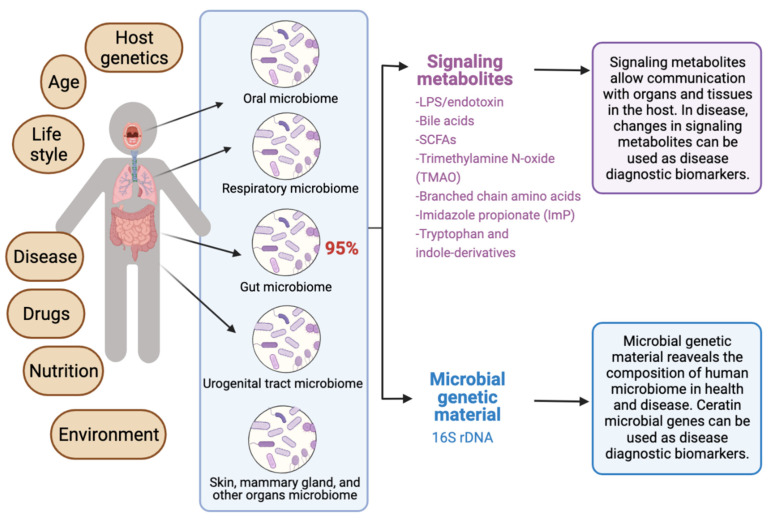
Exploiting the human microbiome for diagnostic disease biomarkers.

Recent reviews indicated that microbiome signatures can be exploited as disease diagnostic biomarkers [71,72,74,75,76,77,78,79]. Herein, we review the available evidence supporting the use of the human microbiome- and microbiota-derived metabolites for the purposes of disease diagnosis. A graphical summary of the concept in provided in Figure 1. We detail potential microbiota-derived biomarkers for the diagnosis of a variety of diseases, including complex diseases like diabetes, neuro-diseases, and cancer.

## 2. The Rationale for Microbiome-Based Disease Biomarkers

The identification of “ideal biomarkers” is considered a daunting task for many diseases, including some cancer types. Most of the current sampling techniques for cancer tissues cannot identify individuals who will lack response to therapy, and they fall short in classifying cancer types correctly, owing to the inter- and intra-tumor heterogeneity of tumors [80]. A biomarker should be easily measurable, non-invasive, and cost-effective. The human microbiome, particularly the gut microbiome, can be considered as a non-invasive approach to identify disease biomarkers that can detect many diseases in the early stages [71,81]. Additionally, the identification of microbiome-based biomarkers can increase the accuracy of disease classification when it is combined with clinical information and other biomarkers. For example, some microbes are known to contribute to the adenoma-carcinoma transition in some cancers, such as colorectal carcer (CRC). Such microbes can be exploited as disease and immunotherapy efficacy biomarkers for CRC [71,81].

In addition to microbiome-based biomarkers, there is also an emerging interest in mast cells (MCs) [82,83,84,85], microRNAs (miRNAs) [86,87], imaging, and machine-learning models [42] as non-invasive disease diagnostic and prognostic biomarkers that promise to shape the future of precision medicine. Sometimes, there is a crosstalk between the human microbiota and other genetic or chemical biomarkers. For example, alterations in fecal small RNA profiles in CRC reflect gut microbiome composition in stool samples [88]. Thus, using multiple connected biomarkers of the network type (i.e., “network biomarkers”) may increase the effectiveness of existing biomarkers.

## 3. The Significance of Human Microbiota in Health and Disease

The human microbiota plays several important roles in the human body, such as helping in food digestion, producing vitamins, regulating the immune system, and protecting against pathogenic disease-causing microbes. In the following subsections, we review the significance of the human microbiota in health and disease and the importance of classifying healthy microbiomes from unhealthy microbiomes in clinical practice.

### 3.1. Conservation of Homeostasis

The human microbiota controls the immune system and affects the inflammatory cascade and immune homeostasis in newborn and children [89]. Children developing allergies at advanced ages showed ubiquity of anaerobic bacteria and Bacteroidaceae, as well as a low number of *Lactobacillus*, *Bifidobacterium bifidum*, and *Bifidobacterium adolescentis* [11,27]. Studies reported that these microbes hydrolyze adulterants such as pesticides, plastic particles, heavy metals, polycyclic aromatic hydrocarbons, and organic compounds [23]. Further studies revealed that the urinary tract microbiomes detoxify toxins [90]. Studies showed that female genital tract microbiomes provoke an immune response through secreting antimicrobial peptides, inhibitory compounds, and cytokines [90]. 

### 3.2. Involvement in Host Immune System

The symbiosis interaction between the indigenous microbiome and the immune system results in the evolution of immune responses and the development of the immune system to recognize pathogens and beneficial microbiota [91,92]. Indeed, the immune system is shaped by the human microbiome [93]. The lack or alterations in the human microbiome might weaken the immune system and induce type II immunity responses and allergies [39,94]. Aberrations of microbiota induce allergic rhinitis in children [39,94]. The gut microbiome activates the regulatory T-cells (Tregs) and proinflammatory Th17cells in the intestine [95,96]. The older neutrophil decreases the proinflammatory properties in vivo [91]. The microbiota induces the growth of neutrophil through MyD88-mediated and Toll-like receptor (TLR) signaling cascades [91]. Changes in microbial flora decrease the old neutrophils and induce inflammation-mediated tissue injury, such as septic shock and sickle cell disease. Altogether, the microbial flora supervise disease-inducing neutrophil, which is a substantial component in inflammatory diseases [91]. In addition, the gut microbiomes protect the body against harmful pathogens through inducing colonization resistance, as well as synthesizing antimicrobial compounds [97]. A stable intestinal microbiota controls antibodies of CD8^+^T (killer) and CD4^+^ (helper) cells that impede the influx of the influenza virus to the respiratory system [89,97]. The gut flora supports and optimizes the functionality of the GIT [98,99]. Activating the regulatory T cells is essential in maintaining the hemostasis of the immune system [89]. 

### 3.3. Involvement in Host Nutrition and Metabolism

Gut microbiota provide nutrients to the host by digesting complex dietary elements (e.g., fiber and other complex carbohydrates) in food, permitting their absorption from the gut [100]. Additionally, intestinal microbiota offer essential nutrients that are not available, but are necessary for maintaining GI tract functionality [101]. Furthermore, intestinal microbiota halt cancer prognosis in the GI tract by generating butyrate, which is a product of fermentation complex nutrients [102]. Studies revealed that fruits’ and vegetables’ carbohydrates maintain a healthy GI tract microbiome [97]. In addition, the gut microbiome provide the required vitamins (K and folic acid) for host growth, such as enterobacteria and GI tract bacteria, including *Bacteroides* and *Bifidobacterium* species [100]. Moreover, gut microbiota contribute to red and white blood cells (RBC and WBC) synthesis [103]. Live microorganisms (probiotics) are deployed for treating allergic diseases [97]. Probiotics decrease and/or inhibit the activation of T-cells and restrain the tumor necrosis factor (TNF) that participates in systemic inflammation [97]. Gut microbiota produce important vitamins needed for blood coagulation, including B vitamins such as B12, thiamine and riboflavin, and Vitamin K [104,105,106].

### 3.4. Classifying Healthy and Unhealthy Microbiomes

The identification of microbiome-based biomarkers for disease diagnosis, prognosis, risk profiling, and precision medicine relies on the determination of microbial features associated with health or disease. It is often a daunting task to clearly define what constitutes a healthy microbiome in different human populations, especially because a person’s microbiota can be affected by many factors, including age, lifestyle, diet, smoking, exercise, ethnicity, environmental factors, and other factors. Another challenge in classifying healthy versus unhealthy microbiomes stems from limitations in the current technologies and methodologies that do not provide a high microbial resolution on the strain-level, impeding the functional understanding or relevance for health or disease [10]. 

## 4. Metagenomics-Derived Genes as Potential Disease Biomarkers

There is emerging evidence highlighting important functional links between microbiota dysbiosis and disease. Cataloging the types of organisms and the numbers of each type is extremely helpful in studying microbial dysbiosis. This is often achieved by metagenomics, the study of the genetic composition (genomes) of a mixed community of organisms recovered from environmental and human samples. Metagenomic studies can be performed using either high-throughput shotgun genomics (i.e., metagenomics sequencing) [107], or by the use of the polymerase chain reaction (PCR), based on 16S rRNA gene amplicon sequencing analysis, to study microbial ribosomal RNA (rRNA) [108,109]. The use of 16S rRNA amplicon sequencing allows the comprehensive phylogenetic assessment of the studied microbiome. However, microbiome researchers are currently using database-independent operational taxonomic unit (OTU)-based methods [110,111,112], which reduce the taxonomic resolution, and impair further functional analysis at the strain level.

## 5. Microbiota-Derived Metabolites as Potential Disease Biomarkers

Gut microbiota-derived metabolites are considered as central regulators in metabolic disorders and are important surrogates to study microbial dysbiosis [113,114,115]. For example, microbial metabolites such as bile acid derivatives, short-chain fatty acids, branched-chain amino acids, trimethylamine N-oxide, tryptophan, and indole derivatives, have been implicated in the pathogenesis of multiple metabolic disorders [115]. These metabolites are considered potential diagnostic and prognostic disease biomarkers, as well as promising targets for drug discovery and development. Both gut and serum metabolomes can be targeted to identify such metabolomics’ biomarkers. Examples on most important bacterial metabolites with biomarker potential in human disease are provided in Table 1, based on data mined from the Human Metabolome Database (HMDB) version 5.0 [116] and the Marker Database (MarkerDB) [117].

### 5.1. Short-Chain Fatty Acids (SCFAs)

These are subclasses of saturated fatty acids that contain six or fewer carbons [119]. They include acetate, propionate, butyrate, pentanoic (valeric) acid, and hexanoic (caproic) acid [120]. CSFAs are the main bacterial metabolites due to an anaerobic fermentation of indigestible dietary fiber and resistant starch by specific colonic anaerobic bacteria in the large intestine [121]. It is currently believed that SCFAs, particularly those of low molecular weights (acetate, propionate, and butyrate), play crucial role in the physiology of various systems, at both the cellular and molecular levels [122]. SCFAs play vital roles in terms of colonic health [123]. It is well established that SCFAs have anti-inflammatory, antitumorigenic, and antimicrobial activity [120]. SCFAs are now evidently involved in the pathogenesis of chronic diseases such as allergies, asthma, cancer, autoimmune and metabolic diseases, and most significantly, neurologic conditions [124]. Fecal SCFAs have the potential to be used as biomarkers for irritable bowel syndrome (IBS) [125]. Serum SCFAs have the potential to be used as biomarkers for multiple sclerosis [126,127] and colorectal cancer [128].

### 5.2. Branched-Chain Amino Acids (BCAAs)

These are essential amino acids whose carbon structure is marked by a branch point and which are obtained directly form sources such as meat, dairy, and legumes. They include leucine, isoleucine, and valine [129]. BCAAs supplementation is believed to have a promoting effect on anabolic pathways and may play an essential role in the protection against muscle wasting (cachexia), chronic kidney disease and liver cirrhosis, attenuating exercise-related fatigue, the promotion of wound healing, and the stimulation of insulin production [130]. BCAAs are considered potential biomarkers for insulin resistance, type 2 diabetes, the risk of cardiovascular disease, stage I and II chronic kidney disease, ischemic stroke [131,132], major depression [133], dyslipidemia [134], and chronic graft vs. host disease [135].

### 5.3. Tryptophan and Indole-Derivative Metabolites

Tryptophan is an essential amino acid that is necessary for normal infant growth, the production and maintenance of the body’s proteins, enzymes, and neurotransmitters [136]. It also plays an important role in regulating the sleep cycle and appetite, as it is a precursor for the synthesis of melatonin and serotonin [137]. Tryptophan is also a precursor of niacin (vitamin B3) [138]. It is found in dairy products, nutritional seeds, white meat, and fish [139,140,141,142,143]. Indole metabolites that are produced via the microbial metabolism of tryptophan include indole-3-propionic acid (IPA) and indole-3-aldehyde (IAld) [144]. These indole derivatives possess anti-inflammatory, antibiotic, antioxidant, and immunomodulatory effects [145]. In fact, the kynurenine/tryptophan ratio has been investigated as a potential blood-based biomarker in non-small cell lung cancer [146], while indole-derived metabolites have been considered as potential indicators for body mass index [147].

### 5.4. Trimethylamine N-Oxide (TMAO)

This is an amine oxide that is produced by the gut microbial metabolism of carnitine and choline. TMAO is evidenced to exacerbate glucose tolerance, inhibit hepatic insulin signaling, and promote inflammation; hence, it is considered as a mediating molecule to develop type-2 diabetes mellitus. Studies also suggest a crucial role of TMAO in the development of atherosclerosis and the pathophysiology of ischemic heart diseases [148]. TMAO has the potential to serve as a novel biomarker for plaque rupture in patients with ST-segment elevation myocardial infarction (STEMI) and early metabolic syndrome [149]; it is also a promising diagnostic biomarker for cardiovascular and neurological disorders [150], as well as for preeclampsia [151].

### 5.5. Imidazole Propionate (ImP) 

This compound is identified as a novel microbial metabolite produced through the alternative metabolism of histidine in type 2 diabetes mellitus patients. ImP may be considered as a potential biomarker for elevated blood pressure in obese patients [152].

### 5.6. Bile Acids

These play an important role in the innate immune defense within the intestine, since they are considered as potent antimicrobials that have an essential role in the defense mechanism of the host microbiota [153]. Both host and microbiota regulate the bile acid pool. The liver bile acid–microbiome axis has been implicated in many diseases, including liver cirrhosis and hepatocarcinogenesis [154,155,156,157]. After bile acids are synthesized in the host liver, they are converted to secondary bile acids by gut microbiota. Reduced bile acid levels in the GI tract are usually associated with bacterial overgrowth and inflammation [158]. High fat diets increase the levels of bile acids in the gut, which affect the highest taxonomic levels of gut bacteria. Physiological concentrations of various intestinal bile acids play an important role in preventing the intestinal colonization by pathogens such as *Clostridium difficile* [159]. Increased bile acids lead to blooms of taxa, including bile acid 7α-dehydroxylating species such as *Clostridium scindens* and *Clostridium hylemonae.*

### 5.7. Lipopolysaccharides (LPS), Lipooligosaccharides (LOS) and Endotoxin 

Lipopolysaccharides (LPS) are macromolecules consisting typically of a hydrophobic domain known as lipid A (or endotoxin), a non-repeating “core” oligosaccharide, and a distal polysaccharide (or O-antigen). They are considered important constituents of the outer membranes of Gram-negative bacteria. The term lipooligosaccharide (LOS) is used to refer to a low-molecular-weight bacterial lipopolysaccharide. Endotoxin (lipid A), the hydrophobic anchor of lipopolysaccharide (LPS), is a glucosamine-based phospholipid that is present in the outer membranes of most Gram-negative bacteria. Gut microbiota-derived endotoxin has been linked to human disease, including GI tract inflammation in Parkinson’s disease [160], nonalcoholic fatty liver disease (NAFLD) [161], and preeclampsia [151]; it is also linked to neurotoxicity [162]. Systemic exposure to bacterial endotoxin can be detected by measuring plasma LPS binding protein (LBP).

## 6. Microbiome Signatures as Disease Biomarkers

The microbial abundance and compositional patterns identified from metagenomic analyses can be used as disease biomarkers. However, the search for such signatures in human cohorts has been confounded by environmental factors, host factors, disease status, and the presence of other comorbidities [108]. Gut microbiome signatures are used as biomarkers for many disease conditions, including central nervous system [43], inflammatory [163], and metabolic disorders [163]. Therefore, these signatures remain as important aspects of the human microbiome regarding the identification of diagnostic biomarkers for human disease.

## 7. Microbiome Multi-Omics

Sequence-based methods relying on 16S ribosomal RNA (rRNA) amplicon sequencing, while very important in identifying microbiome-based biomarkers, provide very limited information on the functional relationships within microbial communities, or between the microbiota and the human host. Therefore, researchers are increasingly combining 16S rRNA analyses with the more costly shotgun metagenomics to obtain functional insight. Shotgun metagenomics allows researchers to comprehensively sample all genes in all organisms present in a given biological sample [164]. Additionally, metagenomic data can be complemented by RNA sequencing, which creates metatranscriptomic profiles for microbial communities that can be used to determine the metaproteomic and metametabolomic profiles of constituent microbial communities. This allows the validation of metagenomic findings by elucidating the mechanisms that link microbial metabolism with various diseases. Thus, metabolomics can be used to examine the crosstalk between the microbiome and the host through metabolites. The level of correlation between taxa at different taxonomic levels and metabolites has been described in the biomedical literature [112]. Additionally, certain bacterial proteins and enzymes, such as nucleases, have shown promise as diagnostic tools and treatments [165]. For example, *Serratia marcescens* nuclease (EC 3.1.30.2) has therapeutic value for the treatment of respiratory diseases, resulting in sputum production due to its ability to hydrolyze sputum DNA effectively [165].

## 8. Association Predictions of Microbiome and Other Omics Data

Multi-omics is a promising approach to predict the diagnosis, prognosis, and treatment efficiency of diseases. Genes, RNA, proteins, metabolites, microbes, and pathways, as well as pathological and medical imaging data, can all be integrated and analyzed comprehensively by means of network analysis to come up with a unified and potentially more accurate hypothesis about the disease in question [166]. Such networks enable the exploration of the relationships between biological entities to determine their function and relevance to the disease. The fusion of multimodal data for cancer diagnosis is considered a feasible research framework for radiomics and genomics [167]. Recently, some clinical trials have used diverse approaches to define characteristics of the patients who develop primary or acquired resistance to immunotherapy (e.g., NCT04243720) [168]. Such trials are aiming to develop an integrated model to predict drug resistance relying on multimodal data including radiomics, genomics, transcriptomics, epigenetics, immunophenotypic data, and fecal microbiome data [80]. There is promise that artificial intelligence models combining microbiome-based biomarkers with other omics data (e.g., radiomics) will be able to provide a more comprehensive view of the tumor microenvironment, aiding in better cancer diagnosis and allowing clinicians to non-invasively track changes in cancer phenotypes [80].

## 9. Diseases which Can Be Probed Using Microbiome-Based Biomarkers

Changes in the normal microbiota have been linked with different diseases such as cancer, inflammatory bowel disease, neuro-disease, cardiovascular disease, systemic infections, allergic diseases, and others. Table 2 summarizes diseases that can exploit the microbiome for diagnostic biomarkers. Important major condition groups are discussed thoroughly in the following sections. These major groups include cancer, central nervous system diseases, inflammatory bowel diseases, cardiovascular diseases, allergic diseases, and systemic infections.

### 9.1. Cancer

Studies showed that microbiomes perform biochemical reactions affecting cancer prognosis and proliferation, as well as immunotherapy reactions [16,24]. Recurrent GI tract infections and antimicrobial drugs are linked to dysbiosis and colorectal cancer [24]. The metabolites of gut microbiota affect the intestinal lining, inducing or inhibiting carcinogenesis [33,102]. Gut microbiota contribute in colorectal cancer and hepatocellular carcinoma [33,102]. Additionally, *Clostridium*, *Fusobacterium*, and *H. pylori* contribute in gastric cancer [33]. Studies showed that *E. coli* induce lung cancer cell movement, adherence, and metastasis through Toll-like receptor 4 (TLR4) signaling via suppressing TLR4 (Eritoran), p38 mitogen-activated protein kinases (MAPK), and extracellular signal-regulated kinase (ERK1/2) phosphorylation [266]. Females with breast cancer, as opposed to healthy females, showed *Staphylococcus*, *Enterobacteriaceae*, and *Bacillus* in breast tissues [267]. *Lactobacillus* species were absent in the breast cells of breast cancer females. Moreover, *Escherichia coli* and *Staphylococcus epidermidis* were detected in cervical cancer [267]. Prostate cancer patients showed higher frequencies of *Bacteroides massiliensis* [61,62].

Studies showed that *Streptococcus* and *Veillonella* infections in airway epithelial cells are modulated through phosphoinositide 3-kinase (PI3K) and extracellular signal-regulated kinase (ERK) signaling cascades [268]. Further studies revealed that *Acidovorax*, *Comamonas*, *Klebsiella*, *Rhodoferax*, and *Polarmonas* are linked to lung squamous cell carcinoma (LUSC), having tumor protein p53 (TP53) mutations [269]. Furthermore, studies reported that the pulmonary microbiome mediates lung cancer prognosis by inducing myeloid-cell-dependent interleukin (IL) (IL-1β and IL-23) and activating lung-resident T cells (Vγ6 + Vδ1 + γδ T cells) [270]. Researches showed that smoking mediates *Acidimicrobiales norank*, *Caulobacteraceae*, and *Enterobacter* spp. infection [271,272] in the lower respiratory tract, altering the respiratory and immunity response mechanisms, such as the dendritic *cells* (DCs), natural killer (NK) cells, macrophages, immunological memory (T and B) lymphocytes, CD8^+^, CD4^+^, and CD25^+^ Tregs. Studies showed that cigarette smoking incites pulmonary cell membrane damage, facilitating cancer proliferation and bacterial transfer to lung cancer [273]. Epidemiological studies recorded that *Chlamydia pneumoniae*, tuberculosis (TB), mycoplasma, and pneumococcal infection increase the risk of lung cancer [274,275,276,277,278].

Studies declared that bacterial, fungal, and viral infections are risk determinants for cancer prognosis. Particularly, 15% of cancer cases evolved by oncogenic organism infection [279], and other cases emerged by co-infection with diverse pathogens that promote the risk of cancer development [279]. Therefore, the investigation of infection-mediated cancer is necessary to impede cancer prognosis and enhance treatment protocols.

Studies showed that hepatitis B (HBV) and C viruses (HCV), (5%), human papilloma viruses (HPV) (5%), Helicobacter pylori (5%), Epstein–Barr virus (EBV) (1%), human immunodeficiency virus (HIV) (1%), human herpes virus (HSV) (1%), helminth (*Schistosoma haematobium*), and fungi (*Aspergillus* spp.) are implicated in cancer development [60,67,68,280,281]. Viruses-mediated cancer can be contracted in uterus, during adulthood, or during childhood; however, these viruses have long incubation periods prior to cancer induction. Moreover, the liability to infectious diseases is excessive in cancer patients [64].

Furthermore, cancer treatment can alter the host microbiome because of immunocompromising activity, thus enhancing infection liability and consequently, cancer prognosis [64]. Eventually, cancer microbiota has been linked to chemotherapy resistance [64]. Studies reported that the gut microbiome can affect the effectiveness of anticancer treatment, such as oxaliplatin [71].The intestinal microbiome recruits the myeloid cells for generating high concentrations of reactive oxygen species (ROS). The high levels of ROS induce oxaliplatin-accompanied DNA deterioration and consequently, promote cancer cell death [71].

Alternatively, cyclophosphamide, an alkylating chemotherapeutic agent, damages the epithelium of the small intestine and subsequently modulates anticancer activity [282]. Biological studies showed that the efficacy of 5-fluorouracil (5-FU) decreases in cells invaded by *Mycoplasma hyorhinis* due to bacterial thymidine phosphorylase that transforms anticancer drugs [283]. Further studies revealed that bacteria can deactivate gemcitabine due to bacterial cytidine deaminase [283]. Studies showed that the detection of *Faecalibacterium* spp. in gut the microbiome of melanoma patients was accompanied by the anti-programmed cell death 1 (PD-1)/PD-L1 response, whereas *Anaerotruncus colihominis, Bacteroides thetaiotaomicron*, and *Escherichia coli* were accompanied by the absence of such a response [284].

### 9.2. Irritable Bowel Syndrome and Inflammatory Bowel Disease (IBD) (Crohn’s Disease and Ulcerative Colitis)

Evidence suggests the existence of various pathogenic factors contributing to irritable bowel syndrome and inflammatory bowel diseases, including genetic predisposition, chronic low-grade intestinal inflammation, personality traits, and microbiome alterations [285]. Studies focusing on microbiome alternations indicated that microbiota dysbiosis invoked irregular immune reactions against body cells and tissues, resulting in autoimmune, GI tract inflammatory, and other threatening diseases [97]. A steady beneficial relationship is established between the human microbiota and the immune system. A disturbance in this relationship weakens the host’s immunity, resulting in an abnormal inflammatory response [93], such as an inflammatory bowel disease (IBD) [89]. A decrease in GI tract Firmicutes increases proinflammatory cytokines (IL12, IFN-γ) and decreases anti-inflammatory cytokine (IL-10) [89]. Reported studies demonstrated that helminth infections accompany anti-inflammatory microbes that impede IBD progress in mice models [280].

### 9.3. Cardiovascular Diseases

The gut microbes produce trimethylamine N-oxide (TMAO) metabolites that might contribute to cardiac disease [31,281]. Diets containing phosphatidylcholine, choline, and l-carnitine are transformed by hepatic monooxygenase to trimethylamine, which consequently metabolizes to trimethylamine N-oxide (TMAO). TMAO disrupts lipid transportation and invokes precursor production that induces atherosclerosis and artery thickening [31,281]. Clinical studies showed that disturbances in intestinal microbes are observed in cardiovascular disease patients [18,103]. Further studies showed that hypertensive patients have higher levels of *Prevotella* and *Klebsiella* in the stool. Additionally, hypertensive mice demonstrated a substantial increase in Firmicutes to Bacteriodetes ratio in the stool [18,103].

### 9.4. Systematic Infections

The translocation of microbes increases the probability of systemic disease in immune-deficient patients [103]. The translocated microbes generate uremic toxins, activating the inflammatory response and inducing diseases [103,286]. The misuse of antibiotics and impairment of gut epithelium induce the proliferation of anaerobic microbes and weaken the immune response [103,286]. A disturbance in GI tract microbes promotes the synthesis of nitrogenous compounds that affects the epithelial structure and in turn, facilitates the movement of microbiota and their toxins to other locations in the body [286]. Clinical studies showed that hemodialysis patients have translocated gut microbiota, implying a relationship between kidney disease and gut microbiota [31,281].

### 9.5. Allergic Diseases

The mucosal lining of the respiratory system is affected by gut microbiota. A disruption in the gut microbiome has an impact on the lung microbiome through microaspiration, which increases the risk of respiratory diseases [44]. Clinical studies reported that neonatal Caesarean delivery potentiates allergic diseases due to lack of maternal flora [94]. Further biological studies showed that Caesarean-delivered children have low levels of Bacteriodetes, or normal flora, in the GI tract [18]. Studies declared that a decrease in Bacteriodetes anti-inflammatory activity is accompanied by asthma and rhinitis [97]. Studies recorded that there is a substantial connection between the disruption of the microbiome and allergic antigens (IGE) [287]. Studies showed that children with lower levels of *Akkermansia*, *Bifidobacterium*, and *Faecalibacterium* are sensitive to numerous respiratory allergies that might lead to asthma at around 4 years of age [288]. Studies revealed that residing in a farming environment, with a variety of microbial consortium, is accompanied with a lower rate of respiratory allergies [289,290]. Earlier evidence showed that growing mice in a “farm dust” environment with a diverse bacteria community weakens the respiratory allergic response [46]. Further proof declared that exposing mice to *Acinetobacter lwoffii* F78 and *Lactococcus lactis* G121 shows a protective effect against respiratory inflammation [291].

## 10. The Clinical Implications of Using Microbiome-Based Biomarkers

The development of microbiome-based diagnostic biomarkers is considered one of the key aspects of precision medicine [17,292]. A large body of evidence highlighted an important role of human microbiota in modulating health and disease, through many immune and non-immune mechanisms, via changes in RNA, DNA, and metabolite networks. For example, inflammation, genotoxicity, and metabolism are fundamental mechanisms to modulate carcinogenesis by microbiota, and can therefore be exploited to develop personalized anticancer therapies [17].

However, most of the currently-available evidence linking the human microbiome to cancer and other non-inflammatory bowel diseases is considered preliminary or limited [293,294]. Therefore, there is a great need for more in vitro and in vivo confirmatory tests to prioritize reliable microbiome-based diagnostic biomarkers, drug targets, or personalized treatments [17]. The identification of validated predictive microbiome-based biomarkers could revolutionize the field of precision medicine by guiding clinical decision making about disease diagnosis and proper personalized treatments.

## 11. Challenges and Future Direction

The development of valid clinical biomarkers and the generation of curated microbial genetic databases are becoming essential tools for disease diagnosis and pharmacotherapy monitoring. Additionally, understanding the regulatory, microbial contamination, and safety protocols regarding microbiome bench work is important to speed up the translation of basic research into clinical interventions [295]. Many challenges in microbiome research are related to method standardization concerning biological variation [296], diet [297], complex chemical backbones [298], access to in vivo sampling locations [299], time intervals [300], collaboration of financial and human resources [301], biodiversity and clinical aspects [302], and interactions with body tissues [303]. Additionally, the determination of microbial features associated with health versus disease requires improved microbiome profiling technologies, with strain-level resolution which is still unattainable [10].

Another challenge is the difficulty in establishing the clinical importance of inter-individual differences in the gut microbiome, since some parts of the microbiome (e.g., uncultured bacteria, viruses, phage, fungi, and archaea) are poorly characterized, invoking scientists to refer to them collectively as microbial “dark matter.” Al Bataineh et al. [5] has explored the intestinal fungal dark matter, and found evidence of microbiota involvement in host metabolism and aging pathways.

Microbiome research requires novel strategies for the standardization and mechanistic validation of the identified microbial gene clusters. Integrated multi-omics methods— combined with cataloging bacterial isolates, profiling metabolites, and measuring host responses—have permitted the correlation of bacteria and bacterial metabolites with numerous diseases. However, we are now faced with new challenges concerning the causal relationships of the human microbiome in context with normal physiology and disease pathways [304,305,306]. Revolutionary research is needed to understand the causal relationships between human microbiota and human disease by understanding the underlying mechanisms through which microbes affect human health. Such understanding would advance microbiome research beyond biomarker validation to identify therapeutic drug targets. In fact, bacterial metabolites can provide colossal mechanistic insight that may accelerate the development of new therapeutic strategies for various diseases, such as the management of impaired glucose metabolism in diabetes [307]. Furthermore, the inclusion of detailed host demographical data, such as age, sex, ethnicity, geography, dietary habits, exercise, and other factors, could help in the identification of personalized diagnostic biomarkers.

## 12. Conclusions

The human microbiota will pave the road for a new era in biomarker research for disease diagnosis and pharmacotherapy monitoring. This will ultimately revolutionize the field of precision medicine and individualized treatments. However, more collaborative work is needed to develop robust, comprehensive, and open-source databases powered by novel methodologies that allow researchers across the world to upload, explore, visualize, and interpret their data, and also standardize their methods to be able to compare their results with those of other research groups.

## Figures and Tables

**Table 1:** Important microbiota metabolites that can be explored as diagnostic biomarkers.

Metabolite (HMDB ID) ^a^	Biospecimen Location ^b^	Associated Diseases and Disorders ^c^	Diseases with Abnormal/Diagnostic Levels (Clinical Status) ^d^
Acetic acid/Acetate (HMDB0000042)	Blood Breast Milk Breath Cerebrospinal Fluid (CSF) Feces Saliva Urine	Argininosuccinic aciduria Asthma Autism Celiac disease Colorectal cancer Crohn’s disease Diverticular disease Early preeclampsia Enteritis Eosinophilic esophagitis Gout IBS Lung cancer Maple syrup urine disease Multiple sclerosis NAFLD Pervasive developmental disorder not otherwise specified Phenylketonuria Prediction of fetal disorder/disease during pregnancy Propionic acidemia Sepsis Tyrosinemia Ulcerative colitis	Argininosuccinic aciduria (investigational) Maple syrup urine disease (investigational) Preeclampsia/eclampsia (investigational) Pregnancy (investigational) Propionic acidemia (investigational)
Propionic acid/Propionate (HMDB0000237)	Blood Cerebrospinal Fluid (CSF) Feces Saliva Urine	Autism Celiac disease Colorectal cancer Crohn’s disease Eosinophilic esophagitis IBS Methylmalonic acidemia NAFLD Pervasive developmental disorder not otherwise specified Propionic acidemia Rheumatoid arthritis Ulcerative colitis	Crohn’s disease (investigational) Eosinophilic esophagitis (investigational) IBS (investigational) Methylmalonic acidemia (investigational) Propionic acidemia (investigational) Ulcerative colitis (investigational)
Butyrate (HMDB0000039)	Blood Breast Milk Breath Cerebrospinal Fluid (CSF) Feces Saliva Urine	Asthma Crohn’s disease Ulcerative colitis Colorectal cancer IBS NAFLD Celiac disease Autism Pervasive developmental disorder not otherwise specified Bladder infections Diverticular disease Rheumatoid arthritis Enteritis AIDS Eosinophilic esophagitis	AIDS (investigational) Crohn’s disease (investigational) (investigational) IBS (investigational) Ulcerative colitis (investigational)
Valeric acid/Pentanoic acid (HMDB0000892)		Asthma Autism Bladder infections Celiac disease Colorectal cancer Crohn’s disease Diverticular disease Eosinophilic esophagitis IBS Metastatic melanoma NAFLD Pervasive developmental disorder not otherwise specified Ulcerative colitis	Celiac disease (investigational)
Caproic acid/Hexanoic acid (HMDB0000535)	Blood Cerebrospinal Fluid (CSF) Feces Saliva Sweat Urine	Autism Celiac disease *Clostridium difficile* infection Colorectal cancer Crohn’s disease IBS Medium Chain Acyl-CoA Dehydrogenase Deficiency NAFLD Pervasive developmental disorder not otherwise specified Ulcerative colitis	Celiac disease (investigational) Medium chain acyl-CoA dehydrogenase deficiency (investigational)
Isoleucine (HMDB0000172)	Blood Breast Milk Cerebrospinal Fluid (CSF) Feces Saliva Sweat Urine	Alzheimer’s disease Autism Autosomal dominant polycystic kidney disease Colorectal cancer Crohn’s disease Dementia Eosinophilic esophagitis Epilepsy Heart failure IBS Leukemia Lewy body disease Maple syrup urine disease Pancreatic cancer Periodontal disease Phenylketonuria Preeclampsia Pregnancy Rheumatoid arthritis Saccharopinuria Schizophrenia Ulcerative colitis	Alzheimer’s disease (clinical) Autosomal dominant polycystic kidney disease (clinical) Maple syrup urine disease (clinical) Heart failure (clinical)
Leucine (HMDB0000687)	Blood Breast Milk Cerebrospinal Fluid (CSF) Feces Saliva Sweat Urine	Alzheimer’s disease Autism Colorectal cancer Crohn’s disease Dementia Eosinophilic esophagitis Epilepsy Heart failure IBS Leukemia Lewy body disease Maple syrup urine disease Pancreatic cancer Periodontal disease Phenylketonuria Preeclampsia Pregnancy Rheumatoid arthritis Schizophrenia Ulcerative colitis	Alzheimer’s disease (clinical) Heart failure (clinical) Maple syrup urine disease (clinical)
Valine (HMDB0000883)	Blood Breast Milk Cerebrospinal Fluid (CSF) Feces Saliva Sweat Urine	Alzheimer’s disease Autism Autosomal dominant polycystic kidney disease Cachexia Colorectal cancer Crohn’s disease Dementia Diabetes mellitus type 1 Diverticular disease Eosinophilic esophagitis Epilepsy Gout Heart failure IBS Leukemia Lewy body disease Maple syrup urine disease Obesity Pancreatic cancer Paraquat poisoning Periodontal disease Phenylketonuria Preeclampsia Pregnancy Rheumatoid arthritis Schizophrenia Ulcerative colitis	Alzheimer’s disease (clinical) Cachexia (clinical) Maple syrup urine disease (clinical) Paraquat poisoning (clinical)
Imidazole propionate/ImP (HMDB0002271)	Blood Feces Saliva	Colorectal cancer Supragingival plaque	Not available
LPS with O-antigen (HMDB0013470)	Blood	Not available	
LPS core (HMDB13471)	Blood	Not available	
Trimethylamine N-oxide/TMAO (HMDB0000925)	Blood Cerebrospinal Fluid (CSF) Feces Saliva Urine	Argininosuccinic aciduria Celiac disease Colorectal cancer Crohn’s disease Dimethylglycine dehydrogenase deficiency Eosinophilic esophagitis Inflammatory bowel disease Kidney disease Lung cancer Maple syrup urine disease Pancreatic cancer Propionic acidemia Rhabdomyolysis Schizophrenia Trimethylaminuria Tyrosinemia I Ulcerative colitis Uremia	Kidney disease (clinical) Uremia (clinical) Maple syrup urine disease (investigational) Argininosuccinic Aciduria (investigational) Uremia (investigational) Lung cancer (investigational) Trimethylaminuria (investigational) Dimethylglycinuria (investigational)
Tryptophan (HMDB0030396)	Urine	Eosinophilic esophagitis	Leukemia (clinical) Alzheimer’s disease (clinical) Eosinophilic esophagitis (clinical)

^a^ HMDB ID: Human Metabolome Database (HMDB) [118] ID; ^b^ according to the HMDB 5.0 [116]; ^c^ according to the HMDB 5.0 [116]; ^d^ levels and development status designation according to the Marker Database (MarkerDB) [101].

**Table 2:** Diseases that can exploit the human microbiome and microbiota metabolites as diagnostic biomarkers.

Disease	Evidence of Microbiome Involvement
Acne	[169,170,171,172]
Allergic Rhinitis	[173,174]
Alzheimer’s Disease	[175,176,177,178]
Amyotrophic Lateral Sclerosis	[179,180,181]
Ankylosing Spondylitis	[182,183]
Anxiety Disorders	[184,185,186]
Asthma	[187,188,189]
Atopic Dermatitis	[190,191,192]
Autism Spectrum Disorders	[193,194,195,196]
Behcet’s Disease	[197,198,199]
Breast Cancer	[200,201]
Cardiovascular Disease	[202,203]
Chronic Constipation	[204,205]
Coronaviruses	[206,207,208]
Depression	[209,210]
Diabetes	[211,212]
Diarrheal Diseases	[213,214,215]
Epilepsy	[216,217]
Fibromyalgia Syndrome	[218,219]
Fungal Infections	[220]
Headache Disorders	[221]
HIV and AIDS	[222,223]
Inflammatory Bowel Disease (Crohn’s Disease and Ulcerative Colitis)	[224,225]
Irritable Bowel Syndrome	[226,227]
Lung Cancer	[228,229]
Melanoma	[230,231]
Metabolic Syndrome	[232,233]
Multidrug-Resistant Bacterial Infections	[234,235]
Multiple Sclerosis	[236,237]
Myalgic Encephalomyelitis/Chronic Fatigue Syndrome	[238,239]
Neurologic Cancer	[240,241]
Nonalcoholic Fatty Liver Disease	[242,243]
Obesity	[244,245]
Pain	[246,247]
Pancreatic Cancer	[248,249]
Parkinson’s Disease	[250,251]
Phenylketonuria	[252,253]
Psoriasis	[254,255]
Rheumatoid Arthritis	[256,257]
Rosacea	[258,259]
Transplant Rejection	[260,261]
Tuberculosis	[262,263]
Wound Healing	[264,265]

## Abbreviations

5-FU	5-Fluorouracil
BCAAs	Branched-Chain Amino Acids
CD4+	Cluster of Differentiation 4 Positive
CD8+	Cluster of Differentiation 8 Positive
DCs	Dendritic Cells
CSF	Cerebrospinal Fluid
EBV	Epstein–Barr Virus
ERK	Extracellular Signal-Regulated Kinase
GI	Gastrointestinal
HBV	Hepatitis B Virus
HCV	Hepatitis C Virus
HIV	Human Immunodeficiency Virus
HMDB	Human Metabolome Database
HPV	Human Papilloma Virus
HSV	Herpes Simplex Virus
IBS	Irritable Bowel Syndrome
IAld	Indole-3-aldehyde
IGE	Immunoglobulin E
IL	Interleukin
ImP	Imidazole Propionate
IPA	Indole-3-propionic Acid
LBP	LPS Binding Protein
LOS	Lipooligosaccharides
LPS	Lipopolysaccharides
LUSC	Lung Squamous Cell Carcinoma
MAPK	Mitogen-Activated Protein Kinases
NAFLD	Nonalcoholic Fatty Liver Disease
NK	Natural Killer
OTU	Operational Taxonomic Unit
PCR	Polymerase Chain Reaction
PD-1	Programmed Cell Death 1
PI3K	Phosphoinositide 3-Kinase
RBC	Red Blood Cells
ROS	Reactive Oxygen Species
rRNA	Ribosomal RNA
SCFAs	Short-Chain Fatty Acids
TB	Tuberculosis
TLR	Toll-Like Receptor
TLR4	Toll-Like Receptor 4
TMAO	Trimethylamine N-Oxide
TNF	Tumor Necrosis Factor
TP53	Tumor Protein p53
Tregs	Regulatory T-Cells
WBC	White Blood Cells
